# Turning over New Leaves

**Published:** 1890-06-21

**Authors:** 


### Turning Over New Leaves.

### A City Girl.*

The lady who chooses to be known as "John Law" is the true historian of Whitechapel. Mr. Besant and Mr. Gissing are sensitive enough to the sordid setting of life at the East-end, but John Law knows that life to the core. His costermonger and his sempstress are not idealised out of knowledge; they are not humorous, save unconsciously; neither are they conscious of the pathos of their fate. They are not clever, their views of life are limited, and they do not go yearning after finer issues; but they are uncompromisingly real, unmistakably human. The story of "A City Girl" is a simple one enough, the "old tale and often told" of the working girl whose love wanders from an uncultured sweetheart to be wasted on a gentleman; the old story of disillusion and repentance; and in most respects Nelly, the heroine, is commonplace enough. But the other characters are fresh, and the surroundings are new.

In one sense we might say painfully new, for the scene of the story is one of those new blocks of workmen's buildings which, with the best intentions, philanthropic people are running up all over the metropolis. John Law does not seem to think much of them, neither does he appear to set great store on many things which we have been accustomed to regard as useful, and which are certainly intended to modify the hardships of the lives of the poor. The lady rent-collector does not meet with much approval, and the hospital sister here depicted is so unamiable a character that we are glad the author has felt obliged to protest in a footnote against his readers accepting her as a type of her class. Here is her portrait:—

> She was a pretty woman, this sister; a little widow, who had taken to nursing because she was poor—because she thought a nurse held a better position in the eyes of the world than a governess. She knew that many rich ladies try to get rid of *ennui* in hospitals, but no rich ladies make the same experiment in school-rooms. She had relations in the House of Commons, a cousin in the House of Lords, and connections in all the professions, more especially, she was wont to say, in the army. She was always afraid that people were forgetting her rank, was on thorns about her social position—and she had reasons for this, most likely, considering the odd dips the social scales give when people have large pretensions and little money. She spoke to "a great lady" with a lisp, almost with a drawl, in quite a different voice from the one she used when talking to patients or nurses, even to doctors.

We confess to having met nurses who had some of the characteristics of this sister, but we are happy to think that they are rare. Still, we do not get a pleasant picture of hospital matters in this book; even the villain of the piece is the treasurer of an East-end hospital. This gentleman can, however, be easily relegated into the category of things invented; but there are two hospital scenes which have the unmistakable stamp of truth. One is where poor Nelly surprises the nurses by asking them if any of them has had a baby, and can hardly be persuaded to leave her own baby to the care of childless women. The baby died, and the sister already depicted takes Nelly to see it in the mortuary. Here most people would have pictured a touching scene of maternal grief, touched with gratitude for the charity that surrounded death with beauty and the symbols of hope. Not so John Law, and we cannot help thinking the conduct he ascribes to Nelly is true to the life:—

> She opened her mouth and showed the astonished sister what it is to be an East-end mother. She made the West-end lady shiver and shake, for she vowed that the nurses had killed her boy, and she swore the most terrible oaths against childless wives and unmarried women who dared to call themselves nurses. Her bonnet dropped on the ground, her red-brown hair fell over her shoulders, her hazel eyes glistened as she shook her fist at the sister.
>
> "Take that!" she said, and with one blow she knocked the childless nurse to the ground.

Captain Lobe, the hero of one of John Law's previous stories, comes into this, and shows the high estimate in which the author, who, as we have said, speaks with knowledge, holds the work of the Salvation Army. There is a rather comic scene between Captain Lobe, full of "the Army," and the commands of "the General," and George, the ex-soldier, Nelly's sweetheart, who knows no army but the Queen's. George is in many respects the freshest character in the book. We must sympathise with him in the regret that rises to his lips as he encounters one after another of the problems of independent life: "I wish I'd never left the service." In the service he had had no vote; he had been forbidden to marry; his work had been fixed; and, like many other people, he had found obedience easier than self-rule. There is something new, too, in the sweater with a back-ache, the sweater who worked from four in the morning till eleven or twelve at night. He is not a pleasant character, and his wife is more unpleasant still; but that back-ache disarms one. How would it be if the sweaters went on strike for an eight-hours day? Altogether "A City Girl" presents a picture of East-end life more accurate than those to which we are accustomed, and none the less valuable that it shows what flaws may lurk even in the well-intended efforts of our philanthropy.

* "A City Girl." By John Law. (London: Authors' Co-operative Publishing Co., 20 and 22, St. Bride Street, E.C. Price One Shilling.)

